# Predicting DNA damage foci and their experimental readout with 2D microscopy: a unified approach applied to photon and neutron exposures

**DOI:** 10.1038/s41598-019-50408-5

**Published:** 2019-09-30

**Authors:** Sofia Barbieri, Gabriele Babini, Jacopo Morini, Werner Friedland, Manuela Buonanno, Veljko Grilj, David J. Brenner, Andrea Ottolenghi, Giorgio Baiocco

**Affiliations:** 1 Physics Department, University of Pavia, Pavia, Italy; 2 Institute of Radiation Medicine, Helmholtz Zentrum München, German Research Center for Environmental Health, Neuherberg, Germany; 3 Center for Radiological Research, Columbia University Medical Center, New York, USA

**Keywords:** Computational biophysics, Biological physics

## Abstract

The consideration of how a given technique affects results of experimental measurements is a must to achieve correct data interpretation. This might be challenging when it comes to measurements on biological systems, where it is unrealistic to have full control (*e*.*g*. through a software replica) of all steps in the measurement chain. In this work we address how the effectiveness of different radiation qualities in inducing biological damage can be assessed measuring DNA damage foci yields, only provided that artefacts related to the scoring technique are adequately considered. To this aim, we developed a unified stochastic modelling approach that, starting from radiation tracks, predicts both the induction, spatial distribution and complexity of DNA damage, and the experimental readout of foci when immunocytochemistry coupled to 2D fluorescence microscopy is used. The approach is used to interpret γ-H2AX data for photon and neutron exposures. When foci are reconstructed in the whole cell nucleus, we obtain information on damage characteristics “behind” experimental observations, as the average damage content of a focus. We reproduce how the detection technique affects experimental findings, *e*.*g*. contributing to the saturation of foci yields scored at 30 minutes after exposure with increasing dose and to the lack of dose dependence for yields at 24 hours.

## Introduction

Ionizing radiation is capable to induce severe damage to critical cell targets as the DNA and chromosomes. Damage directly associated to the passage of radiation in a cell might arise as a consequence of energy deposition in the DNA macromolecule, in form of discrete ionization or excitation events distributed on a nanometric spatial scale, or be mediated by attack of radicals generated by energy depositions to water molecules in proximity to the DNA. The final fate of a cell then depends on a variety of biological mechanisms activated as a consequence of initial damage induction, notably DNA repair processes. Also, radiation effects can be the result of more complex cascades of events involving non-hit cells and intra- and extra-cellular signalling, and that can be initiated by radiation^1^. Evidently, the large variety of possible pathways and players makes it impossible to formulate a unique mechanistic description of cellular response to the radiation insult. Nevertheless, a mechanistic approach in the description of radiation-induced DNA damage starting from physical interactions has proven to be successful, in particular when it comes to the comparison of the effects of different radiation qualities: different types of radiation (or particles of the same nature but with different energies) lead to different spatial distribution and clustering of energy depositions, resulting in different levels of DNA lesion complexity.

Track-structure codes coupled to a software replica of DNA as a target^2^ are a remarkable example of mechanistic modelling, fully taking into account the stochasticity of energy deposition by radiation, and have been successfully applied to obtain predictions on initial DNA damage and its complexity^3^. Such predictions might constitute the starting point for further modelling steps, usually requiring a phenomenological approach to correlate initial damage to late radiobiological endpoints^4^. A direct benchmark of theoretical predictions with experimental results on DNA damage is also possible, but limited to damage whose characteristics can be measured with the adopted technique and irradiation condition (*e*.*g*. dose level): an example in this sense is the use of PFGE (pulsed field gel electrophoresis) to detect DNA fragments of high molecular weight (~10 kbp to ~10 Mbp) following irradiation with relatively high doses^5^. Specific information on shorter fragments cannot be derived from data obtained using this technique. Conversely, modelling results on the fragment length distribution have been used in^6,7^ to inform PFGE data analysis and interpretation: high-LET radiation leads to a DNA fragmentation pattern with a predominance of very short fragments, hence a non-flat fragment length distribution in the smallest detectable-size interval. If the mid-size of the interval is used to calculate the experimental yield of fragments from the total detected mass, such yield is largely underestimated. When the theoretical average value of the fragment length distribution in the same small-size interval is used, a very good agreement is found with the predicted fragment yield.

Applications of modelling based on radiation tracks to DNA^4,8–10^ or chromatin fragmentation (in case of chromosomal aberrations^11^) offer a proof of the potential of this approach to go “behind” experimental observations, delivering insight on damage characteristics that are “unmeasurable” with the chosen technique. Fully exploiting this potential, we can implement a strategy to further simulate how the damage appears “to the eye of the observer”, namely simulate the readout with the chosen experimental technique. Conceptually, this is equivalent to what is done when the functioning of a measurement device is fully simulated, and theoretical predictions on the desired observable are “filtered”, so that a direct comparison with acquired experimental data becomes possible. Obviously, when it comes to measurements on biological systems, it is simply unrealistic to imagine a software replica of the whole measurement chain, that involves (on top of biological processes) complex laboratory procedures, use of a wide variety of instruments and large operator intervention. Nevertheless, considering in the modelling how the specific aspects of a measurement technique influence the readout of a chosen endpoint might greatly assist in the interpretation of radiobiological data (in particular when the aim is to compare the effectiveness of different radiation fields), as we discuss in this work for the case of DNA damage foci.

Foci arise as a consequence of the re-localization of proteins involved in the DNA damage response system^12^, which is activated when the cell identifies lesions to the DNA as those induced by exogenous agents as ionizing radiation^13^. One of the early events associated with the sensing and processing of DNA DSBs (double strand breaks) is the phosphorylation of the H2AX histone^5,14,15^ (then called γ-H2AX)^16,17^, a variant of one of the core proteins (H2A) forming the octamer around which DNA is tightly wrapped in the structural unit called nucleosome^18^. The phosphorylation of H2AX acts as a platform for the recruitment of repair factors (as well as checkpoint proteins and chromatin remodelling complexes)^19–21^, causing their accumulation at the break site, leading to the formation of ionizing radiation induced foci. The phosphorylation is not limited to the first neighbour histones, but it spreads over a region of the order of the ~Mbp size^5^, acting as a signal amplifier and efficiently communicating the presence of the DSB. It persists (with self-reinforced accumulation of repair factors^22–24^) as long as the repair is ongoing, and dephosphorylation occurs at the end of the process, so that the cell can recover from checkpoints and progress in the cycle^25^. This results in a kinetics of foci formation and disappearance after the initial insult by radiation^26^.

Experimental information on DNA DSBs can therefore be derived detecting foci, whose presence indicates that DNA repair processes are ongoing^27^. Phospho-specific antibodies that detect phosphorylated targets (the H2AX histone in this case) can be used *e*.*g*. in western blot, flow cytometry, immunocytochemistry (ICC) coupled to fluorescence microscopy. Time-series measurements should be planned to characterize the whole kinetics associated with DNA damage foci induction and disassembly/removal. Among the possible detection techniques, only microscopy can be used to quantify the γ-H2AX signal in terms of yields of foci, potentially correlated to the underlying number of DNA DSBs^28,29^. This however translates into an extremely time-consuming analysis, given the large number of images that has to be acquired and processed for each condition (dose-point, time-point and radiation qualities, when more are compared). This number is further increased when using a *Z*-stack technique or resorting to confocal microscopy, to detect foci in the whole nucleus instead of using conventional 2D microscopy. Advanced microscopy techniques^27,30^ also reveal substructures within DNA repair foci, that can help understanding foci formation and associated biological pathways.

Most importantly, there are intrinsic limitations to be taken into account, that might hinder a proper quantification of the insult to the DNA by means of this endpoint: (i) as mentioned, foci have an intrinsic physical extension, associated to the size of the region in the genome that is interested by the phosphorylation at the site of damage; (ii) images acquired with the microscope have a finite resolution, that translates into the possibility of neighboring foci to be unresolved; (iii) when using conventional microscopy, only foci in a single slice at focus in the whole nuclear volume are considered, and the 2D image is obtained by their projection onto a 2D plane, which also leads to possible foci superposition and merging^31^. All these physics- and geometry-related factors contribute to the saturation in foci yields as a function of increasing dose that is observed experimentally^32–34^. Additionally, we need to consider that dense energy deposition by high-LET radiation results in many close damage sites that can concur to the formation of a single focus^35,36^. As well known, all this implies that it is not possible to assume a 1:1 correspondence between foci yields and underlying DSBs.

It is also important to mention that the use of live cell imaging and image tracking algorithms (instead of fixed cell assay at fixed time points) allows to characterize not only the temporal, but also the spatial evolution of DNA damage, providing experimental evidence of the mobility of DNA damage sites. In budding yeast and Drosophila DSBs are clearly mobile and preferentially repaired in specific region of the nucleus^37^, but evidences on the existence of repair domains in human cells are also accumulating. Repair domains are themselves claimed to be either fixed or free to explore the nuclear domain^38^, and seem to be located near the boundary between high and low density DNA regions^39^. Therefore, foci motion and possible merging over time should be also considered as a factor of biological origin making a thorough quantification of the damage even more difficult. Recent findings^40^ on 53BP1 foci induced by radiation in primary skin fibroblasts derived from different strains of mice also indicate that strain differences are preserved under various experimental conditions and also influence signal saturation as a function of increasing dose: this suggests that the specificity of the DNA repair system (number and sizes of repair domains) depends on the genetic background and affects signal saturation.

Taken all together, these considerations lead to the conclusion that great care should be used in the interpretation of radiobiological data on γ-H2AX foci, in particular if the aim is to draw conclusions on the radiobiological effectiveness of a given radiation field based on this endpoint. Therefore, there is need of solid methodologies and criteria to interpret data on foci yields to derive information on DNA damage.

The idea at the basis of this work is that, as in the above-mentioned examples, modelling approaches based on radiation tracks coupled to the simulation of the “observer” can provide essential information to interpret data on DNA damage foci, particularly helping in recognizing readout artefacts of a “more physical” origin and related to the scoring technique. To this aim, we developed a unified modelling approach to predict the induction of foci following exposure to different radiation qualities, as well as how their readout is affected when performing experiments with ICC techniques followed by conventional 2D microscopy for image acquisition. The novelty of this study resides in the implementation of all these aspects in a unified modelling framework. Dedicated radiobiological measurements of γ-H2AX foci have been performed at RARAF, Columbia University, for the benchmark and test of this approach: cells have been exposed to X-rays, as a reference low-LET spectrum, and to a mixed neutron – photon field available at the facility, with neutrons characterized by an energy distribution similar to what expected in Hiroshima at 1.5 km from the epicentre. We take full advantage of two different well-established Monte Carlo codes: the transport code PHITS^41^ for the characterization of neutron-induced secondary charged particles delivering energy to biological samples; the biophysical track-structure code PARTRAC, for the calculation of initial DNA damage following irradiation with photons and charged particles. As discussed in previous works^42^, the coupling of these two codes successfully allows predictions of DNA lesions following neutron exposure. Starting from DNA DSB and DSB cluster induction in irradiation setups (hence also considering damage of different complexity), the new approach further relies on geometrical considerations to reconstruct foci and how they appear in 2D images. The above-mentioned limiting factors associated with foci detection are considered and translated in geometrical parameters with a physical interpretation. Varying the parameters within a reasonable range of values, the approach assists the interpretation of experimental findings, such as the saturation of foci yields scored at 30 minutes after exposure with increasing dose, and the lack of dose dependence for foci yields scored at 24 hours after exposure. An extension of the approach for the 3D reconstruction of foci in the whole nucleus is also presented, and turns out to be necessary to integrate results that could be obtained from the acquisition and analysis of foci with 3D microscopy techniques. As later discussed in great detail, the mechanistic modelling we propose is based on radiation-induced initial DNA damage only, and does not take into account DNA damage evolution in time and space after induction, as well as other biological factors (among which specific cell genetic content, cell cycle phase, genetic background playing a role in the DNA damage response). In this sense, our approach is not aimed at achieving a quantitative reproduction of experimental data, but still provides necessary information (complementary to biological considerations) to avoid mistaken conclusions on the relative effectiveness of the investigated radiation fields.

## Methods

### Cell cultures and reagents

TS/A mouse mammary adenocarcinoma cells were cultured at 37 °C in a humidified atmosphere with 5% CO_2_ in Dulbecco’s Modified Eagle Medium (DMEM, Gibco) with 10% FBS in T75 flasks (Falcon) up to 90% confluence. Cells used for experiments were kept in culture up to passage 13, at maximum. The day before irradiation, 10^5^ cells in 0.5 ml medium were plated in a 4-chamber Culture Slide flasks (Falcon) in duplicates.

### Irradiation setup

**Figure 1:**
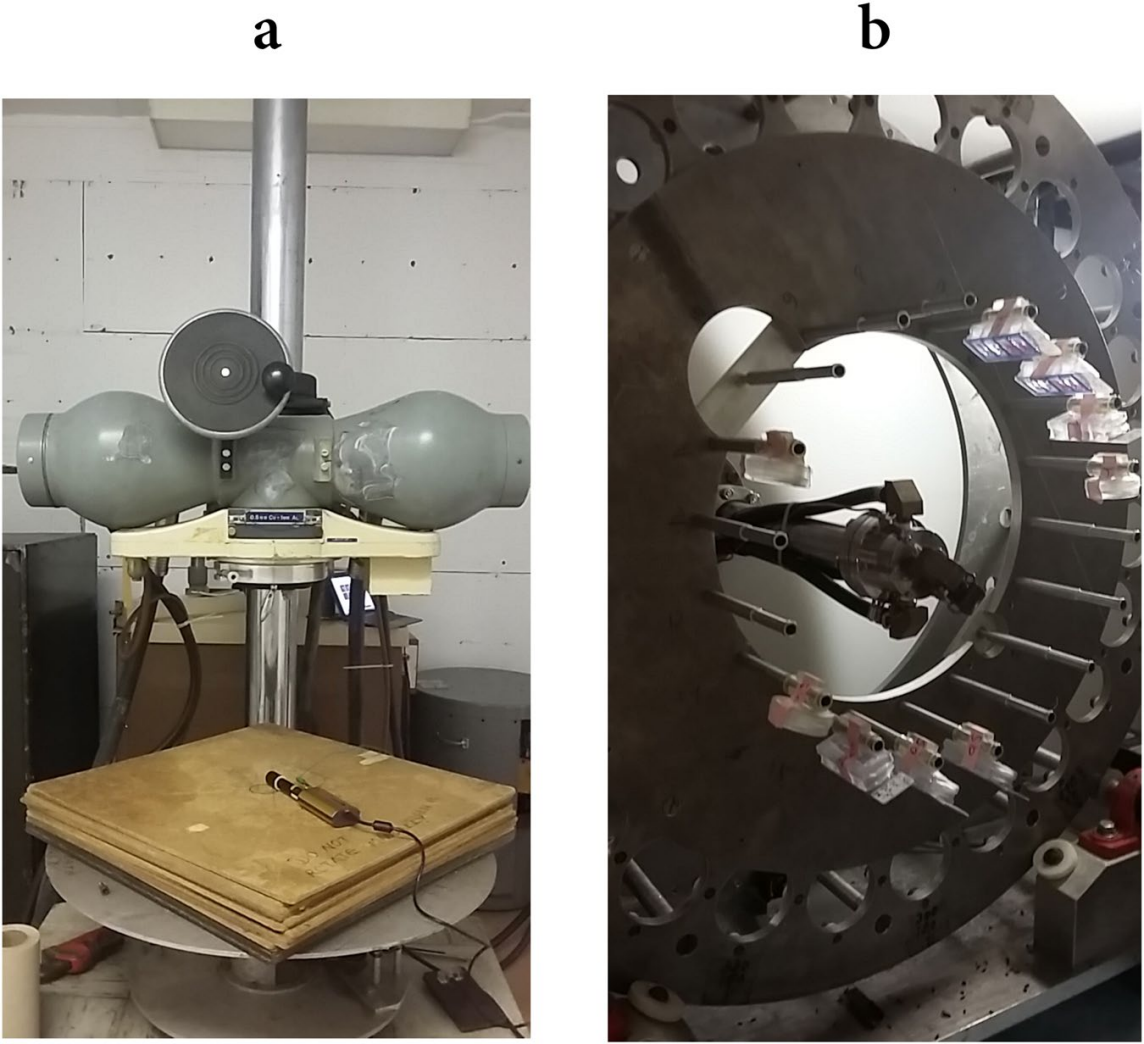
Experimental irradiation setups. Setups for biological sample exposure to X-rays (**a**) and to the mixed neutron – photon field (**b**) at RARAF. The photon beam is perpendicular to the flask surface, the dose rate is of ~1.1 Gy/min. In the mixed field, neutrons (average energy of 2.4 MeV, maximal energy of ~10 MeV, energy distribution referred to as *Hiroshima* neutron spectrum) and photons are generated by a primary 5 MeV mixed proton and deuteron beam impinging onto a Be foil. Flasks are secured to the rods of a metallic wheel, rotating around the primary beam axis, and positioned at a forward angle of 60° and at a distance of 10 cm from the ^9^Be target. The dose rate is of ~0.05 Gy/min, neutrons account for ~83% of the total dose in the mixed field.

Irradiations were performed at the RARAF facility, Columbia University, with cells cultured in Slide flasks. The Westinghouse Coronado X-ray machine (225 kVp, 1 mm Al and 0.5 mm Cu filters) was used for X-ray irradiations. The photon beam is emitted perpendicularly to the flasks. Total doses were of 0 (sham), 1, 2 and 5 Gy, with a dose rate of ~1.1 Gy/min. A picture of the setup is shown in Fig. 1a.

Neutron irradiations were performed with the broad-energy beam described in^43,44^: the neutron field is obtained from ^9^Be(p,n) and ^9^Be(d,n) reactions induced by a 5 MeV mixed proton and deuteron beam from a Singletron linear accelerator, impinging onto a 1-mm thick beryllium foil^45^. Neutrons are produced with a maximal energy of about 10 MeV. Experimental measurements for neutrons above 0.1 MeV give an average energy of 2.4 MeV for the spectrum. The produced neutron field can be considered an analogue of what expected in Hiroshima at 1.5 km from the epicenter^45^ and is referred to as *Hiroshima* neutron field in this work. Neutrons in the field are responsible for ~83% of the total dose delivered to the samples, the remaining ~17% being due to photons generated by the primary charged particle beam impinging onto the ^9^Be target. The overall resulting radiation field is referred to as *mixed neutron – photon field* in this work. The setup is shown in Fig. 1b: the metallic “Ferris” wheel has multiple rods to secure tubes containing biological samples or small animals. The wheel is rotating around the primary beam axis, to assure homogeneity in dose deposition. Slide flasks were positioned at a forward angle of 60° and at a distance of 10 cm from the ^9^Be target^45^. The highest achievable dose rate (~0.05 Gy/min) was adopted to minimize irradiation time. Irradiations were performed for total doses of 0 (sham), 0.1, 0.5, 1 Gy. The highest dose of 1 Gy was delivered in two consecutive 0.5 Gy irradiations, with a time between each dose fraction of less than 3 minutes, much shorter than the total irradiation time.

Experiments were carried out in biological triplicates, each of them comprising a technical triplicate, to verify the reproducibility of the experimental findings.

### Immunocytochemistry and image acquisition

Cells fixation was carried out 30 min and 24 h post-irradiation using 2% paraformaldehyde (Electron Microscopic Sciences) in phosphate buffered saline (PBS, Gibco), at room temperature (RT) for 20 min. For long exposures (as in case of high neutron doses), time is counted from the moment samples are put back in the incubator, in accordance to what done for the other biological samples.

Foci have been visualized by means of fluorescent staining through the following protocol: permeabilization was performed with 100% methanol for 20 min at −20 °C. Cells were washed thrice (5 min each) in PBS and they were blocked in 0.2% bovine serum albumin (BSA, Invitrogen) in PBS at RT, for 15 min. This was followed by 1-h-long incubation with 1:500 anti-γH2AX Rb primary antibody (Cell Signaling Technology) in 0.2% BSA/PBS, at RT. After washing as before, incubation with 1:1000 of anti-rabbit goat IgG Alexa Fluor 555 conjugated secondary antibody (Invitrogen) in 0.2% BSA/PBS was carried out, for 45 min at RT. After washing, sample were mounted and nuclei counterstained using the VectaShield Mounting Medium with DAPI (Vector Laboratories, Inc.).

Image acquisition was carried out using a conventional fluorescent microscope (Olympus IX70 IX-ILL 100 LH), magnification of 60X was used to acquire several fields, to analyze at least 200–250 cells per slide, trying to exclude fragmented or pleomorphic cells.

### Image analysis with ImageJ

2D images were analyzed by means of an ImageJ^46^ semi-automatic macro developed on purpose. Images for the DAPI and γ-H2AX foci channels were analyzed separately: they were first converted to 16-bit and underwent histogram-based binarization (conversion in black and white, to distinguish objects from background) by applying Otsu thresholding^47^, which could be manually adjusted. The analysis then proceeds through application of a smoothing mask, and allows discretionary steps, like holes-filling, volume-dilatation or Watershed segmentation (to separate neighboring objects touching each other), to further refine the reconstruction of the regions of interest (ROIs). For the selection of foci, the steps were the same, except for the adjustment of background levels and the application of the “Unsharp Mask” option, which adds a high-pass filtered image and sharpens the image. Both nuclei and foci were selected by setting thresholds on morphological parameters, *i*.*e*. the circularity (defined in ImageJ as: *4π*^⋅^*area/perimeter*^2^) and the size. For nuclei, the following thresholds were chosen: 5000 < size < 10000 pixel (1 pixel here equal to a square surface element of side 0.15 μm) and 0.8 < circularity < 1. The recognition of ROIs for foci required specific care: a larger variety in size can be expected, as smaller foci might be generated by low-LET with respect to high-LET radiation. The size range 14–750 pixel was chosen, giving a good selection for both cases. The condition in circularity was dropped, to include the possibility of foci with a streak-like structure, as it can be expected for short-range charged particles accelerated in the nucleus. Halos from foci attributed to possible out-of-focus planes were not included in the analysis while thresholding.

Selection criteria entering the ImageJ macro were optimized to reproduce results scored “by eye” by the experimenter for different conditions. For each condition we obtain average results on foci yields per cell. Foci yields are further normalized to results for the sham condition: results on yields are given as extra foci yields (ΔFoci per cell) above background, subtracting foci yields scored in the sham.

Errors on average values are given as standard error of the mean (SEM) for the different biological and technical replicates.

### Neutron transport calculations with PHITS

**Figure 2:**
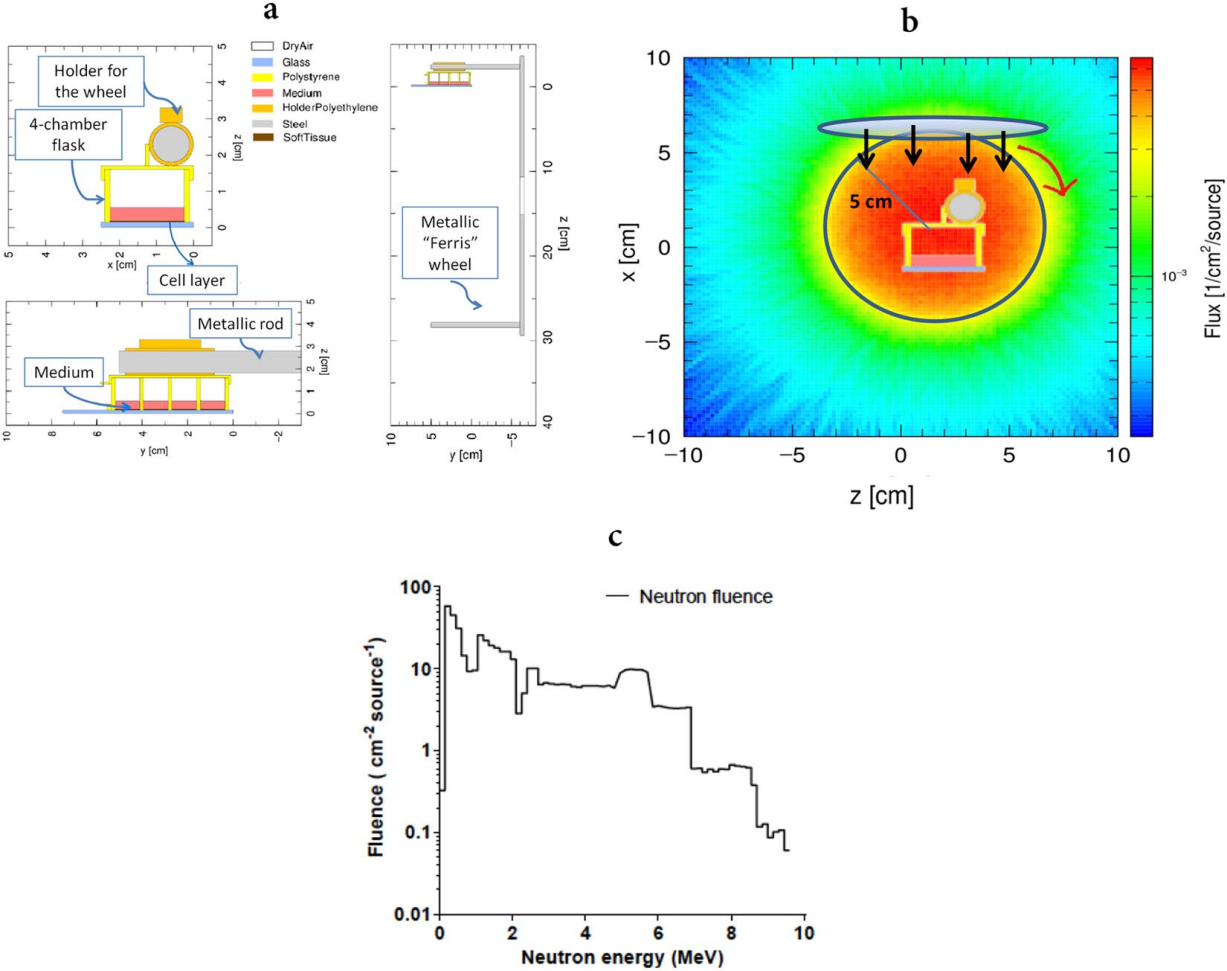
Simulation of neutron irradiation with PHITS. (**a**) Software replica of the experimental setup at RARAF with indication of components and materials: cell layers are reproduced in ICRU 44 tissue and constitute the scoring region for all quantities in the simulation. The Slide flask (glass and polyethylene, containing culture medium), the metallic rod of the wheel and the polyethylene holder are reproduced. (**b**) Irradiation setup implemented in PHITS to reproduce the rotation of the wheel holding the Slide flasks. (**c**) *Hiroshima* neutron energy spectrum (in particles cm^−2^ per source particle) in the volume comprising the flask and the metallic rod, where the biological samples are placed.

We used PHITS (Particle and Heavy Ion Transport code System, v. 3.02)^41^ to characterize the mixed particle field produced in the biological samples following exposure to the *Hiroshima* neutron field. A simplified software replica of the setup used for the irradiations was implemented, including realistic material composition, and it is shown in Fig. 2a. Biological samples (cell layers) were reproduced in the 4-well flask as ICRU 44 tissue^48^ volumes, and they correspond to the scoring region for all quantities of interest in the simulation. Culture medium and air interfaces were also taken into account. Instead of reproducing the wheel rotation, we implemented a neutron source in form of a disk, rotating around a spherical volume comprising the slide flask and the metallic rod (as shown in Fig. 2b). Neutrons are emitted perpendicularly from the rotating disk, and the disk and the spherical volume have the same radius of 5 cm, resulting in an isotropic neutron field at the position of the samples. The energy distribution of neutrons at emission is taken from measured data in^45^, and the resulting spectrum in the volume around the flask and the rod is reported in Fig. 2c.

PHITS transports neutrons from the source and simulates all their interactions with materials, finally recording the characteristics of secondary charged particles delivering energy to the scoring region. We considered secondary protons, α particles, ^12^C, ^14^N, ^16^O nuclei and electrons. Following the approach already adopted in^42^, from the simulation results we extracted two main quantities for each species *s*:

- the relative dose contribution *D*_*s*_ to the total neutron dose. It has to be recalled here that the contribution of photons produced by neutron interactions is scored in terms of dose depositions by electrons, in turn accelerated by secondary photons;
- the dose mean lineal energy ${\bar{{\rm{y}}}}_{{\rm{D}},{\rm{s}}}$ in a sensitive site of 1-μm diameter, as an indicator of radiation clustering properties.

The contribution to the dose to the samples due to photons produced by primary beam interactions with the ^9^Be target for neutron production is not considered in the simulation and later included in the analysis.

Results are average values over 5 simulation runs, each with 10^9^ neutrons emitted from the source, and errors are given as standard deviation of the different PHITS runs.

### Track-structure simulations with PARTRAC

For the simulation of DNA damage we used the biophysical code PARTRAC^49,50^. PARTRAC is a well-established tool, able to simulate radiation-induced DNA damage of different types and complexity, due to energy depositions in the DNA macromolecule both by particle tracks (direct effects) and by free radicals, that are in turn produced by radiation interactions with water molecules in proximity of the DNA (indirect effects).

The spatial distribution of DNA damage can be simulated in a single cell model, whose nucleus contains a software replica of the whole human genome in atomic resolution (~6.6 Gbp) for a lymphocyte- or fibroblast-like cell in its G0/G1 state.

The lymphocyte-like cell model was used to reproduce TSA cells: the model has a spherical nucleus of 10 μm of diameter, while the cytoplasm was simulated as a box centered at the origin of the coordinate system, with sides of 14 μm along the *x* and *y* axes and a thickness along z of 10.2 μm (−5.1 ≤ *z* ≤ 5.1 μm).

PARTRAC allows simulations with photons and charged particles in a wide energy range: as reference low-LET radiation, we implemented as source the spectrum of X-rays generated by a 220 kVp machine (2 mm Cu filter). Photons were generated randomly from the bottom surface of the cytoplasm, emitted parallel to the *z* axis, and calculations were stopped when the dose to the nuclear compartment was reached (1, 2 and 5 Gy), as previously done in a pilot study from our group^51^.

To simulate neutron-induced damage, we performed PARTRAC calculations for secondary charged particles generated by neutron interactions. Given the dominant contribution to the neutron dose, only protons, ^12^C and ^16^O ions were considered for track-structure calculations. As previously discussed, we derived information on these particle characteristics from PHITS simulations. For each species, we then adjusted the irradiation setup in PARTRAC and the starting energy of the particle such that, in the cell nucleus, the average LET corresponds to the dose mean lineal energy calculated by PHITS.

Specifically, this was achieved with the following strategies:

- for protons, irradiating the cell with 0.6 MeV particles emitted by a disk with a surface of 80 μm^2^, rotating tangentially to the cell nucleus, thus generating an isotropic proton field in the nucleus. The disk emits a fixed number of protons in each simulation run, and the total dose delivered to the nucleus is calculated. The following relation between the dose D and particle fluence Φ holds:
  1 $${\rm{D}}({\rm{Gy}})={\rm{LET}}({\rm{keV}}/{\rm{\mu }}{\rm{m}})\cdot \Phi ({{\rm{\mu }}{\rm{m}}}^{-2})\cdot 0.1602$$
  where the average LET in the nuclear volume is found to have the desired value of ~45 keV/μm;
- for ^12^C and ^16^O ions, the same irradiation setup could not be adopted, as particles with the desired LET (respectively, ~375 and ~350 keV/μm) have a too short range to traverse the whole nuclear thickness. Such heavier particles were therefore generated randomly in the nucleus, and their initial energy has been chosen such that the average LET, simply estimated by the ratio of the particle energy to the length of its track, is equal to the desired values. ^12^C and ^16^O energies giving this agreement were found to be, respectively, 1.2 and 0.875 MeV (both in the low-energy branch of the LET vs. energy dependence)^3^.

For all species, the dose dependence was investigated by increasing the number of particle tracks in the simulation. Proton fluences in the range 0.013–0.125 particles ⋅ μm^−2^ have been used (1–10 particles from the 80 μm^2^ surface), corresponding to doses to the cell nucleus from ~0.1 to ~1 Gy. For ^16^O and ^12^C nuclei, 1 to 15 tracks were generated, corresponding to doses from ~0.2 to 4.8 Gy.

In terms of DNA damage induction, for each condition we considered the following simulation output: (i) the total number of DSBs and DSB clusters, where a cluster is defined as the occurrence of two or more DSBs within a genomic distance of 25 bp^52,53^; (ii) the spatial distributions of both DSBs and DSB clusters. Spatial distributions are given by PARTRAC on a voxelized grid: the genetic material in the nucleus is built by 50 × 50 × 50 nm^3^ cubic elements, each containing ~4.8 to 6 kbp in form of a chromatin fiber. Based on this grid, the code delivers information on the coordinates of each voxel in space and its content in terms of DSBs and/or DSB clusters. How these spatial distributions are further used for the modelling of γ-H2AX foci is described later in this section. Results of DNA damage are average values over 32 to 2028 runs, depending on the specific condition. Errors are given as standard error of the mean among the different PARTRAC runs.

### Clustering algorithms to reconstruct γ-H2AX foci

The input of the clustering algorithms is the spatial distribution of DNA damages (either DSBs or DSB clusters) obtained with PARTRAC for a given irradiation condition (*i*.*e*. fixed particle types and number of tracks). The underlying assumption is that a single DSB will act as a recruitment site for repair proteins and will give rise to a focus. DSB (or DSB cluster) spatial coordinates are identified as the coordinates of the center of the voxel where damage is located. If more than one DSB or DSB cluster is present in a voxel, a single set of coordinates is kept for further analysis, but the information on the number of damages is stored (see later). For the sake of simplicity we will generally refer to DSBs in the following, but the same holds when DSB clusters are considered.

The algorithm to reproduce the read-out of γ-H2AX foci in ICC images taken with 2D microscopy (introduced in^51^, referred to as *2D algorithm* in the following) proceeds through the following steps:

1. a slice of thickness *Δz* centered in the middle of the nucleus is selected, and only DSBs within this slice are kept for further analysis;
2. selected DSBs are projected onto the *x-y* plane, their planar coordinates are stored;
3. a recursive algorithm searches for DSBs falling within a planar distance *r*, referred to as *clustering radius*, and it merges them in a single focus;

To reproduce experimental data, different combinations of *Δz* and *r* were tested. The *slice thickness* is varied in the range 0.5 ≤ *Δz* ≤ 1 μm, the *clustering radius* in the range 0.5 ≤ *r* ≤ 1 μm, chosen in both cases based on reasonable expectations on the depth of field (DOF) of the microscope and on the size of the smallest focus observable in the image (resolution) for the sham condition. Though higher than expectations, an additional *Δz* = 2 μm value is tested to reproduce foci yields scored at 30 minutes after irradiation, as later discussed. The algorithm delivers as results average foci yields per cell.

The algorithm to predict the yield of γ-H2AX foci that would have been observed in the whole nuclear volume (*3D algorithm*) starts from the same set of DSB spatial coordinates, but it recursively searches for DSBs falling within a radial distance *R* (a *clustering radius* in 3D space, again in the range 0.5 ≤ *R* ≤ 1 μm) and merges them in a single focus. At difference with what described before, this is done without selecting a slice of the nucleus and without a planar projection of damage coordinates, *i*.*e*. getting rid of artefacts due to the use of 2D images taken with conventional microscopy. The expected average number of foci per cell and foci DSB multiplicity, defined as the number of DSBs that contributed to the focus formation, are given as results.

**Figure 3:**
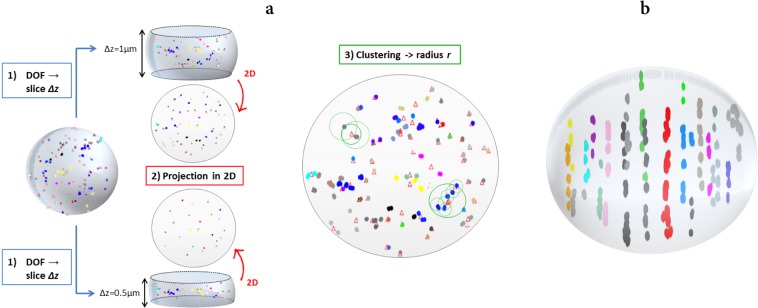
Schematics of the functioning of the clustering algorithm for γ-H2AX foci reconstruction. (**a**) The *2D algorithm* reproduces the read-out of γ-H2AX foci in ICC images taken with 2D microscopy through: selection of a slice of thickness *Δz* (centered in the middle of the nucleus) (1); planar projection of DSBs (DSB clusters) within the slice (2); merging in a single focus of DSBs (DSB clusters) falling within a *clustering radius r* (3). (**b**) The *3D algorithm* reconstructs the yield of foci in the whole nucleus by merging in a single focus DSBs (DSB clusters) falling within a *clustering radius R*.

An illustration further explaining the functioning of the algorithms is shown in Fig. 3.

In this work, results from the *2D algorithm* applied to initial DSB distributions predicted by PARTRAC are used for correlation to experimental 2D-microscopy data on γ-H2AX scored at 30 minutes after irradiation; corresponding results from the *3D algorithm* are used to interpret the data. Results from both algorithms are also applied to PARTRAC initial DSB cluster distributions, and tentatively correlated to data on residual γ-H2AX foci (scored at 24 hours after irradiation) (see the Results and Discussion sections).

## Results

### Experimental results from ICC images and 2D microscopy

**Figure 4:**
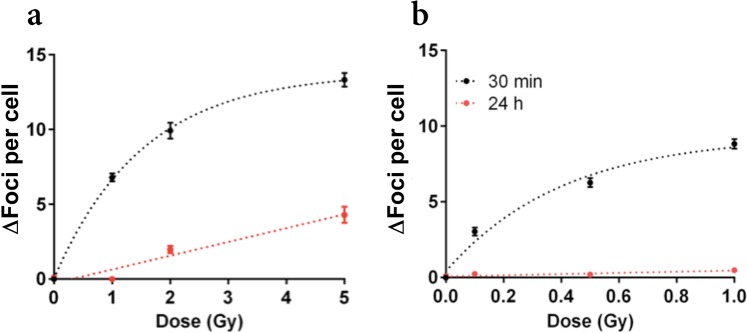
Experimental γ-H2AX foci yields as a function of dose following exposure to X-rays and to the mixed neutron – photon field. Average foci yields above background level (ΔFoci per cell) scored from 2D images at 30 minutes (black) and 24 hours (red) after exposure are shown as a function of increasing dose for X-rays (**a**) and for the mixed neutron – photon field. (**b**) Errors are given as standard error of the mean (SEM) for the different biological and technical replicates. Best fit curves to experimental data are also shown, see the text for further details.

In Fig. 4 foci yields normalized to sham values (ΔFoci per cell) are shown, as scored at 30 min and 24 h after exposure to different doses of X-rays and of the mixed neutron – photon field (panels a and b, respectively). Foci yields scored at 30 min after exposure tend to saturate with increasing dose D, and this is put in evidence by best fit curves shown along with experimental data, obtained with the functional form: ΔFoci(D) = *a* ^⋅^ [1 − exp(−*b* ^⋅^ D)]. The saturation is faster for the mixed neutron – photon field, where the maximum dose (1 Gy) is also lower. Linearity with increasing dose seems to be restored at 24 hours, and data are shown along with best fit curves of the type: ΔFoci(D) = *c* ^⋅^ D. Residual foci yields scored after exposure to the mixed neutron – photon field are barely above background levels also for the maximum dose, which is lower than for the X-ray case.

### Neutron-induced secondary charged particle field in the biological samples

In Table 1 we summarize PHITS results on the characteristics of the secondary charged particles delivering energy to the biological samples following exposure to the *Hiroshima* neutron field. Protons, oxygen and carbon nuclei are all together responsible for more than 98% of the total neutron dose to the samples. Based on this finding, only these particles are further considered for track-structure calculations.

**Table 1:** Characteristics of the *Hiroshima*-neutron-induced secondary charged particle field.

	p	α	C	N	O	e^−^
*D*_*s*_ (%)	87.69 ± 0.30	0.94 ± 0.09	3.03 ± 0.07	0.38 ± 0.01	7.67 ± 0.17	0.13 ± 0.01
${\bar{{\rm{y}}}}_{{\rm{D}},{\rm{s}}}$ (keV/μm)	45.08 ± 0.04	191.10 ± 1.96	374.90 ± 0.50	380.99 ± 4.52	349.89 ± 1.70	2.00 ± 0.01

For each secondary charged species *s*, the relative dose contribution to the total neutron dose *D*_*s*_ (%) and the dose mean lineal energy ${\bar{{\rm{y}}}}_{{\rm{D}},{\rm{s}}}$ (keV/μm) in the biological samples calculated with PHITS. Errors are calculated as standard deviations among different simulation runs.

### DNA damage by photons and neutron-induced secondary charged particles

**Figure 5:**
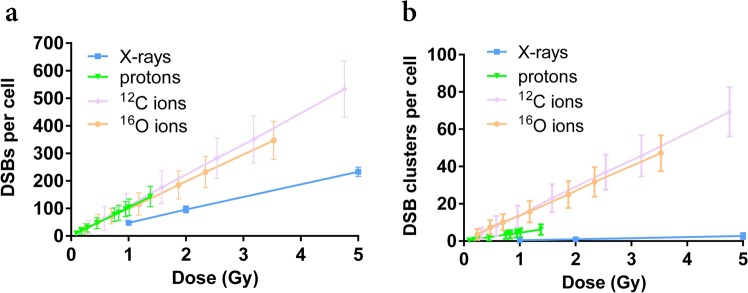
Predicted photon- and charged-particle-induced DNA damage as a function of dose. PARTRAC predictions for DSB (**a**) and DSB cluster (**b**) yields per cell induced by photons (from the reference X-ray field) and secondary protons, ^12^C and ^16^O ions from the *Hiroshima* neutron spectrum. Errors are calculated as standard deviations among different simulation runs. Lines are a guide to the eye.

In Fig. 5 we report PARTRAC predictions for yields of DSBs (a) and DSB clusters (b) as a function of dose, following exposures to photons and to secondary charged particles delivering energy to the samples in the *Hiroshima* neutron field. A linear increase in the damage yields for increasing dose is found for all radiation qualities and for both kinds of damage. Calculations for the reference low-LET spectrum gives, as expected, an average yield of ~45 DSBs Gy^−1^ and less than 1 DSB cluster Gy^−1^. Results for different particles have to be combined to obtain predictions for damage induction by neutrons in the *Hiroshima* field and by the mixed neutron – photon field used for experiments. This is later discussed in this work.

### Predictions of γ-H2AX foci induction and their read-out in 2D images

**Figure 6:**
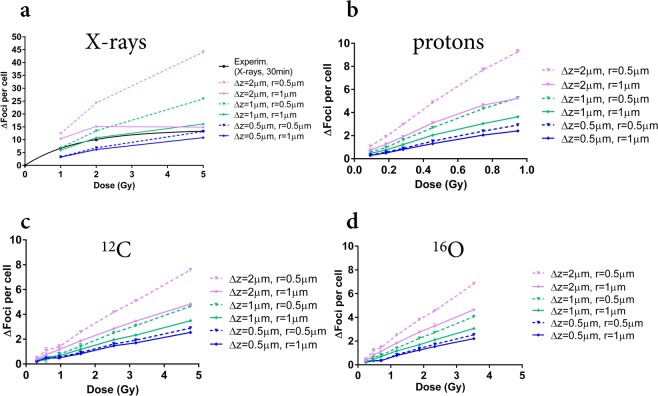
2D γ-H2AX foci read-out. Predictions of the 2D readout of foci yields (from initial DSBs), after exposure to different doses of: photons (from the reference X-ray field) (**a**); secondary protons (**b**), ^12^C (**c**) and ^16^O (**d**) ions from the *Hiroshima* neutron spectrum. The readout is simulated for different combinations of the *slice thickness Δz* (0.5; 2 μm) and *clustering radius r* (0.5; 1 μm) in the 2D clustering algorithm. Errors are calculated as standard deviations among different simulation runs. Lines are a guide to the eye. As a reference, best fit curves to experimental data on foci yields scored at 30 minutes after exposure (Fig. 4) are shown in black for X-rays (**a**).

Figure 6 reports the read-out of γ-H2AX foci yields in 2D images as a function of dose, as predicted by the clustering algorithm applied to the spatial distributions of DSBs. Predictions for different combinations of the *slice thickness Δz* and the *clustering radius r* are shown.

Panel a shows predictions for photon-induced foci, together with the fit to experimental data at 30 minutes after the exposure (corresponding data in Fig. 4). Simulation results show a great variability for the explored range of *Δz* and *r*. At equal dose, lower *Δz* values at fixed *r* imply the removal of a fraction of damages, thus lowering foci yields, though also decreasing the chance of DSBs belonging to different planes being merged after 2D projection. Always at equal dose, lower *r* values at fixed *Δz* lead to a higher probability to distinguish foci arising from DSBs found in close proximity after 2D projection, thus increasing foci yields.

As the dose is increased, the saturation trend observed in experimental data for foci yields is reproduced by different combinations of parameters *Δz* and *r*. More damages are induced, with a higher density in the nuclear volume, and a higher chance of foci arising from single damages not being resolved, thus lowering foci yields.

Panels b–d show predictions for foci induced by protons, ^16^O and ^12^C ions with the same characteristics as secondary particles depositing energy to biological samples in experimental exposures to the *Hiroshima* neutron field. Data for foci induced by the mixed neutron – photon field at RARAF at 30 minutes after the exposure (Fig. 4) cannot be shown alongside with theoretical predictions for secondary species following neutron irradiation only (the dose scale would also be different) and their reproduction requires an additional analysis which is discussed later in this work.

Simulation results for foci yields show less variability with respect to the photon case when different combinations of *Δz* and *r* are considered: this is certainly true for ^16^O and ^12^C ions, and can also be concluded for protons, though calculations have been performed up to a lower maximum dose. This can be ascribed to the fact that damage is more clustered already at induction. As the energy deposited by each proton, ^16^O or ^12^C ion is high, fewer particles are needed to reach the same dose as with the reference low-LET field. Moreover, interactions are still sparse in the nucleus, hence the chance of foci superposition and merging due to projection (hence the role of *Δz*) is reduced. As far as spatial clustering is concerned, foci will have a streak-like morphology, and damages will be so close that, no matter what the value of the *clustering radius r*, many damages will be grouped. In all cases, a maximal increase is observed when the largest value of *Δz* (hence the largest portion of the nucleus) is considered for projection and foci are clustered with the lowest *r*. For ^16^O and ^12^C ions the tracks are short and fully contained in the nucleus, mostly giving rise to single foci.

### Predictions of γ-H2AX foci induction in 3D

**Figure 7:**
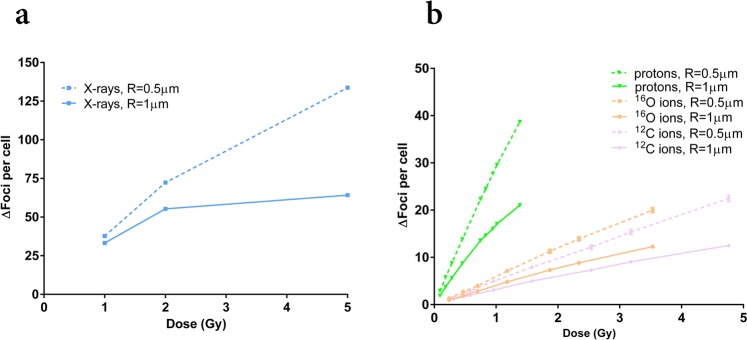
3D reconstruction of γ-H2AX foci yields. γ-H2AX foci yields (from initial DSBs) reconstructed in 3D after exposure to different doses of: photons (from the reference X-ray field) (**a**); secondary protons, ^12^C and ^16^O ions (**b**) from the *Hiroshima* neutron spectrum. Yields are simulated for different values of the *clustering radius R* (0.5; 1 μm) in the 3D clustering algorithm. Errors are calculated as standard deviations among different simulation runs. Lines are a guide to the eye.

Figure 7 reports results of the clustering algorithm applied in 3D for the reconstruction of foci (originating from DSBs) yields in the whole nuclear volume. Results are presented as a function of dose and for values of the *clustering radius R* in the range 0.5–1 μm. Results for X-rays are shown in panel a: comparing to Fig. 5, a deviation from the 1:1 correspondence between the yield of foci and the yield of DSBs is observed for all values of *R*, also at the lowest 1 Gy dose, where we have 33–38 foci per cell (depending on *R*) for 46.9 DSBs. At increasing dose, foci yields tend to saturate, and this is more evident at the larger *R*. Results for protons, ^12^C and ^16^O ions are shown in panel b. In all cases, foci yields show less variability with respect to the photon case when different values of *R* are considered (keeping in mind that proton calculations are performed up to a lower maximum dose). This is coherent with what observed for foci reconstruction in 2D, and can again be ascribed to the fact that damage is more clustered at induction. Again comparing to results in Fig. 5, the yield of foci largely underestimate the total amount of DSBs, as expected in case of high-LET radiation. A slightly saturating trend for foci yields can be guessed for increasing dose, in particular for the largest *R*.

As a strategy to recover the total yield of DSBs starting from the yield of foci, we can use the information on the average number of DSBs contained in a focus (DSB multiplicity). DSB multiplicity values are found to vary as a function of dose and *clustering radius R*. In Table 2 we report for the explored dose range of each species the range of multiplicity values, for both values of *R*.

**Table 2:** DSB multiplicity per focus reconstructed in 3D.

species	dose range (Gy)	*R* = 0.5 μm		*R* = 1 μm	
		min	max	min	max
photons	1–5	1.20 ± 0.06	1.72 ± 0.13	1.37 ± 0.10	3.59 ± 0.77
protons	0.09–0.95	3.10 ± 0.08	3.85 ± 0.08	4.80 ± 0.14	6.06 ± 0.62
^12^C ions	0.31–4.76	13.42 ± 2.33	15.13 ± 2.60	18.65 ± 2.34	27.28 ± 4.53
^16^O ions	0.28–3.53	11.09 ± 1.75	12.85 ± 2.00	16.97 ± 2.13	19.25 ± 3.02

For each species under consideration (photons from the reference X-ray field; secondary protons, ^12^C and ^16^O ions from *Hiroshima* neutron spectrum): maximal and minimal average number of DSBs merged in a single focus (DSB multiplicity), when foci are reconstructed in 3D for different values of the *clustering radius R* (0.5; 1 μm) after exposures in the given dose range.

As *R* is increased, DSBs are scored in a larger volume, hence their multiplicity per focus is also increased. The variation of DSB multiplicity in the explored dose range depends on how sparse damage sites are in the cell nucleus: for the highest doses, damage sites become denser, and foci can be merged also in 3D (in particular for large *R* values). This is coherent with the saturating trends of foci yields shown in Fig. 7.

### Prediction of γ-H2AX foci induction from DSB clusters and correlation to residual damage

**Figure 8:**
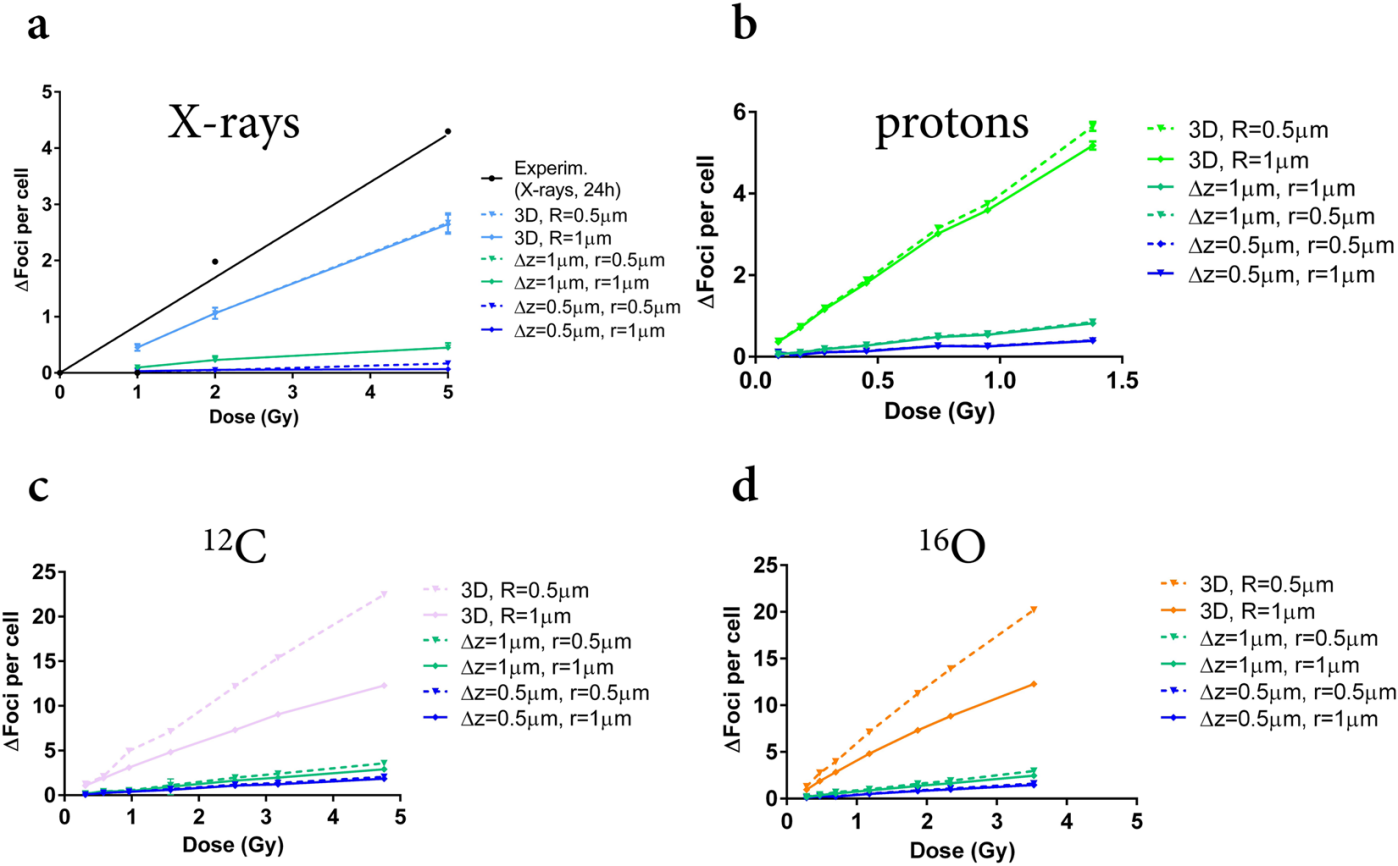
γ-H2AX foci yields from DSB clusters. Predictions of the 2D readout and 3D-reconstructed foci yields from DSB clusters, after exposure to different doses of: photons (from the reference X-ray field) (**a**); secondary protons (**b**), ^12^C (**c**) and ^16^O (**d**) ions from the *Hiroshima* neutron spectrum. Yields are simulated for different combinations of the *slice thickness Δz* (0.5; 1 μm) and *clustering radius r* (0.5; 1 μm) in the 2D clustering algorithm, and for different values of the *clustering radius R* (0.5; 1 μm) in 3D. Errors are calculated as standard deviations among different simulation runs. Lines are a guide to the eye. As a reference, best fit curves to experimental data on foci yields scored at 24 hours after exposure (Fig. 4) are shown in black for X-rays (**a**).

In Fig. 8 we report predictions on γ-H2AX foci yields when the clustering algorithm is applied to the spatial distributions of DSB clusters. The correlation of such predictions to experimental data on residual γ-H2AX foci (scored at 24 h after exposure) is investigated: the underlying hypothesis (later critically discussed) is that only foci originating from damages of a higher complexity (hence, DSB clusters rather than simple DSBs) persist at late time-points. Both predictions of the 2D readout (varying *Δz* and *r*) and of the 3D reconstruction (varying *R*) are shown. In panel a predictions and the linear fit to experimental data (see Fig. 4) for photon-induced residual foci are presented. Foci yields obtained in 2D for all parameter combinations are always close to the background level (0 foci per cell) and do not show a significant dependence with the dose. This is mainly due to the selection of a single slice of the nucleus: only few DSB clusters are induced following photon exposure (less than 1 Gy^−1^), damages are sparse, and there is a high chance that corresponding foci are outside the slice considered for projection. The increase of damage with the dose is instead recovered when all the nuclear volume is considered in the reconstruction of foci in 3D. Experimental foci yields scored in 2D images at 24 h post-exposure increase with the dose above 1 Gy (the experimental point at 1 Gy dose is compatible with zero, but this is not reproduced in the fit, see Fig. 4). Model predictions for foci originating from initial DSB clusters are lower than such yields (with the exception again of the 1 Gy dose point, where the predicted yield is also compatible to zero).

Panels b–d show results for neutron-induced secondary protons, ^12^C and ^16^O ions. As in the case of data for the 30-minute time-point, data for residual foci scored after exposure to the mixed neutron – photon field (Fig. 4) cannot be reported on the same panels. In panel b, similar to what found for photons, proton-induced foci scored in 2D are always close to the zero-per-cell level and no significant dose dependence is predicted, but a net damage increase with the dose is recovered when foci are reconstructed in 3D. Damage induced by secondary protons is more localized along particle tracks, hence a higher number of DSB clusters is induced, but particles are randomly distributed in the nuclear volume and so is the distribution of damages. As for photons, when a single slice is considered for projection, foci arising from DSB clusters can be missed. As previously commented, experimental yields for residual foci scored in 2D are barely above the background level only for the maximum 1 Gy dose, and no significant dose dependence can be seen in the investigated dose range. This seems in good agreement with predictions for proton-induced foci from DSB clusters. For ^12^C- and ^16^O-induced foci only a slight increase with increasing dose is observed in the 2D readout (up to the maximum dose). When applying the reconstruction in 3D, foci yields increase with the dose and show a larger dependence on *R* in the range 0.5–1 μm, which indicate that many DSB clusters are produced in spatial proximity, as expected for short tracks of high-LET particles.

### Coupling transport and track-structure calculations

To obtain predictions on neutron-induced damage, results on the secondary field generated by neutron interactions are to be coupled to results on damage induction by charged particles. This can be done following the approach introduced in^42^ to derive neutron-induced DSB cluster yields as a function of energy, that is here also adapted to foci induction. To obtain predictions on damage induced in the mixed neutron – photon field under investigation in this work, the photon component of such field has also to be considered, and this can be done within the same approach, as described hereafter.

In the following steps, unless specified, the term damage might refer to DSB, DSB cluster yields or foci yields (both from DSB and DSB clusters):

1. the dose delivered by the *Hiroshima* neutron spectrum is decomposed in relative contributions by secondary charged particle species. Only secondary protons, ^12^C and ^16^O ions are considered (the sum of these contributions is normalized to 100%);
2. doses by neutron-induced secondary charged species calculated in step 1. are further normalized, such that their sum gives the neutron-induced component (83%) of the total dose in the mixed field at RARAF, where the remaining dose (17%) is delivered by photons. Final *D*_*s*_ (%) weights for relative doses of particles of species *s* in the mixed neutron – photon field are obtained at this step: *D*_*photons*_ = 17%; *D*_*protons*_ = 74%; *D*_*C*_ = 3%; *D*_*O*_ = 6%;
3. for each species *s*, we obtain results on damage induction (yields) as a function of dose delivered by *s*. For neutron-induced charged particles, this has been done for protons, ^12^C and ^16^O ions having an average LET in the cell nucleus that is equal to the dose mean lineal energy ${\bar{{\rm{y}}}}_{{\rm{D}},{\rm{s}}}$ they have in biological samples exposed to the *Hiroshima* neutron field;
4. DSB and DSB cluster yields are increasing linearly with the dose (see Fig. 5). We can then perform the weighted sum of DSB or DSB clusters induced by photons, protons, ^16^O and ^12^C ions per Gy, using weights *D*_*s*_. This sum gives the yields of DSB or DSB clusters induced by the mixed neutron – photon field at RARAF per Gy per cell;
5. for foci, non-linearity in the yield vs. dose relationship might appear, depending on the radiation type and possibly on the parameters used for the clustering algorithm. At a fixed 1 Gy dose from the mixed neutron – photon field at RARAF, we evaluate the corresponding absolute doses by species *s* using *D*_*s*_ (%) weights given in step 2. Foci (both from DSB and DSB clusters) induced at such doses by photons, protons, ^12^C and ^16^O ions can be calculated using predictions in Figs 6, 7 and 8 for different combinations of *Δz* and *r* when in 2D or different values of *R* when in 3D. When possible, we still use a linear fit to foci yields vs. dose, while when the saturation trend is significant (as it is the case for photon-induced foci yields from DSBs, both in 2D and 3D), we use linear interpolation between pairs of consecutive simulation points (between 0 and 1 Gy for the 0.17 Gy dose contribution by photons to the 1 Gy dose in the mixed neutron – photon field). The sum of these contributions gives the expected foci yields above background per cell exposed to 1 Gy of the mixed field.

Exposure to the mixed neutron – photon field is predicted to induce: 93.3 DSBs Gy^−1^ per cell or 4.6 DSB clusters Gy^−1^ cell. Given the linearity of DSB and DSB cluster yields with dose, relative biological effectiveness (RBE) values can be derived for the induction of these endpoints in the mixed neutron – photon field, by dividing such damage yields by those found in the reference photon field. We thus obtain: RBE (DSB) ~2 and RBE (DSB cluster) ~9.

The 2D readout of foci in cells exposed to 1 Gy of the mixed neutron – photon field is predicted starting from Fig. 6. Results for foci yields from DSBs, both in 2D and 3D, are summarized in Table 3, along with corresponding damage yields and experimental data at 30 minutes post-irradiation; maximum values are obtained when *Δz* = 2 μm and *r* = 0.5 μm. Experimentally, ~9 foci per cell are measured on average from 2D images at 30 minutes after exposure to 1 Gy of the same field (Fig. 4), which falls in the interval of simulated yields. For the same 1 Gy dose of the reference field, the reconstructed 2D readout of foci for *Δz* = 1 μm and *r* = 0.5 μm is ~7 foci/cell, in good quantitative agreement with the experimental finding.

**Table 3:** γ-H2AX foci induction at 1 Gy (data at 30 minutes after exposure and simulations for DSBs).

field (1 Gy)	exp	model		
	2D	2D	3D	DSB yield
photons	7	3–12	33–38	46.9
mixed	9	3–10	18–28	93.3

For the same 1 Gy dose of photons (reference X-ray field) and of the mixed neutron – photon field: experimental results on foci yields at 30 minutes after exposure; range of predicted foci yields (from DSBs) in 2D, varying the slice thickness *Δz* (0.5; 2 μm) and the *clustering radius r* (0.5; 1 μm); range of predicted foci yields (from DSBs) in 3D, varying the *clustering radius R* (0.5; 1 μm).

For exposures to a 1 Gy dose of the mixed neutron – photon field, a 3D reconstruction in the whole nuclear volume (Fig. 7) leads to a predicted foci yield (always from DSBs) in the interval ~18 to 28 per cell, respectively with *R* = 1 or 0.5 μm. For the same dose of the reference field we reconstruct a foci yield in the range ~33 to 38, respectively with *R* = 1 or 0.5 μm.

Results for predicted foci (2D, 3D), when the reconstruction is applied starting from DSB clusters (Fig. 8), are summarized in Table 4, along with corresponding damage yields and experimental results for residual foci.

**Table 4:** γ-H2AX foci induction at 1 Gy (data at 24 hours after exposure and simulations for DSB clusters).

field (1 Gy)	exp	model		
	2D	2D	3D	DSB cluster yield
photons	0	~0	~0.5	0.5
mixed	0	0.3–0.6	3.2–3.6	4.6

For the same 1 Gy dose of photons (reference X-ray field) and of the mixed neutron – photon field: experimental results on foci yields at 24 hours after exposure; range of predicted foci yields (from DSB clusters) in 2D, varying the slice thickness *Δz* (0.5; 1 μm) and the *clustering radius r* (0.5; 1 μm); range of predicted foci yields (from DSB clusters) in 3D, varying the *clustering radius R* (0.5; 1 μm).

In particular, for 1 Gy of the reference field we obtain results always compatible to the background level of zero foci/cell in 2D, and an average yield of ~0.5 foci in 3D, while yields for 1 Gy of the mixed field are higher.

## Discussion

A unified approach to predict DNA repair foci and their experimental readout with 2D microscopy is presented in this paper. The approach is applied to assist the interpretation of radiobiological data for the γ-H2AX endpoint, measured from 2D images of TS/A cells, acquired with conventional fluorescence microscopy from samples treated with ICC techniques. Cells were exposed to a reference 225 kVp X-ray field and to a mixed neutron – photon field at RARAF (see Fig. 1 for pictures of the experimental setups). Neutrons are accounting for 83% of the total dose in the mixed neutron – photon field, the rest of the dose being delivered by photons, and are characterized by the energy distribution shown in Fig. 2 (referred to as the *Hiroshima* neutron spectrum).

A necessary premise for the discussion of results is that the aim of this study was not a quantitative reproduction of the specific dataset here presented, for several reasons we now detail.

First, it has to be kept in mind that simulations of initial DNA damage are themselves affected by uncertainties. We used PARTRAC, a well-established Monte Carlo code that has been benchmarked with experimental data in several works^6,50,54^, mainly considering as an endpoint DNA DSBs measured detecting DNA fragments in given size ranges. Data themselves are affected by experimental errors, possible biases, and influenced by the detection technique, giving a relative wide range of results in terms of DSB/Gy per cell yields in the range 25–45. On top of that, a reasonable uncertainty is associated to the quantitative reproduction of data: as an example, in a most recent work^3^ PARTRAC simulations seem to overestimate experimental DSB/Gy/GBp yields (for pairs of DSB sites that produce DNA fragments in the size interval 5 kbp–6 Mbp) only by about 10–20%. Interestingly, in terms of RBE, simulations do reproduce the measured trends in LET-dependent DSB yields almost perfectly. Different codes (as Geant4-DNA^55^) also give DNA damage predictions that are different, due to differences in some of the hypotheses underlying the modelling, but which, up to now, have proven to be overall comparable, as codes use similar methodology and the same experimental measurements as references.

This has to be taken into account when moving from the “simpler” prediction of total damage to the prediction of damage spatial distribution, as required by the modelling approach presented in this work.

Most importantly, data on γ-H2AX foci (as well as, generally speaking, data on DNA fragmentation) necessarily depend on several biological factors, that are not accounted for in PARTRAC and in our modelling, as *e*.*g*.: the specific genomic content and associated heterogeneity (in particular for cancerous cells) in the cell line adopted for the measurements; the distribution of cells in different phases of the cell cycle at the time of the irradiation. In the modelling instead, foci and their characteristics are predicted based on a single cell model, with human genomic content, in its G0/G1 state. Ideally, a software replica of the specific cell line considered for the experimental measurements could be developed, though this is far from being devoid of difficulties. Taking into account inter-cellular heterogeneity in the implementation of nuclear models for cancerous cells soon becomes unrealistic. It has also to be recalled that measured data most often represent averages on cell populations, which suggests that a modelling approach based on more general features might be preferred. In this work, the software cell model was chosen because of its spherical shape, starting from the observation that cultured TS/A cells have a roundish shape, and based on the hypothesis that geometry is the crucial factor in the simulation of foci experimental readout with 2D microscopy. With this choice, the modelling approach is also challenged in a case (quasi-spherical vs. ellipsoidal nucleus) where we expect (again, based on pure geometrical considerations) that the issue of foci superposition upon 2D-projection becomes of greater importance.

However, the dependence of our results on the cellular model used for the simulation is likely not the most important factor: biological processes activated in response to DNA damage play a major role and might have a larger impact in quantitative terms. Experimental data were obtained at 30 minutes and 24 hours after exposure: based on a full characterization of foci kinetics, the 30-minute time-point was found to correspond to the maximum yield of foci scored from 2D images. A set of simulation of initial damage of the type DSB is used for correlation with experimental data at this time-point. The 24-hour time-point was chosen as an indicator of residual damage, to derive information on biological effectiveness at the end of DNA-repair processes. In this case, we used simulation of initial DSB cluster, under the hypothesis that foci originating from damages of a higher complexity will persist at late time-points. It has to be mentioned here that complex DNA damage can be of various types. We here used the definition of DSB cluster as a DNA lesion comprising at least 2 DSBs within 25 bp, closely following the definition of DSB++, given in^52,53^ and already used in several works^3,42^. Different definitions could have been adopted (as *e*.*g*. a DSB plus a SSB or base damage). Alternative choices would have obviously resulted in different initial damage spectra, and possibly led to different assessment of radiation effectiveness.

In all cases, DNA repair mechanisms and the spatial and temporal kinetics associated to damage repair after initial induction (which, as known, also depends on the specific cell type) are not accounted for in the modelling. Experimental measurements of DNA damage usually take place when DNA repair might have already been initiated, even if cooling samples prior the irradiations might prevent (or slow-down) the initiation of repair pathways. It is important to notice that, in this work, this has a larger impact on the analysis of data from irradiation with the mixed neutron – photon field: the lower dose rate with respect to the X-ray reference field (0.05 vs. 1.1 Gy/min) implies that longer times are needed to achieve the same dose levels, and that foci readout is affected by repair processes presumably ongoing during the irradiation (at least as far as the fast component of the DSB repair is concerned, which has a half-time of 10–30 min^56–58^).

With all these limitations, the approach we propose still delivers important information on how readout artefacts of a “more physical” origin (mainly related to the scoring technique) might affect foci data, and therefore assists in their interpretation, as we discuss in the following. In summary, the approach relies on: neutron transport calculation with PHITS; DNA damage calculations with the track-structure code PARTRAC; and on a 2D clustering algorithm (illustrated in Fig. 3) to go from the spatial distribution of DNA DSBs/DSB clusters to a simulated readout of foci yields. The algorithm is based on geometrical parameters to take into account at the same time the physical extension of foci (hence of the region in the genome interested by the phosphorylation) and all technique-related artefacts that affect the experimental readout, in particular: the selection of a single slice at focus in the nucleus; the projection onto a 2D plane; the final resolution of the image. All these factors possibly concur to the superposition and merging of foci originating from different damage sites. The necessity to develop an approach of this kind stems from a common experimental observation: the γ-H2AX signal (average yields of foci per cell above background levels in our case) saturates for increasing dose, particularly for the earliest time-points after exposures. In the dataset presented in this work, this is observed at 30 minutes after irradiation, both for the reference low-LET field and for the mixed neutron – photon field (with the onset of saturation at lower doses in this latter case) (see Fig. 4). Foci signal saturation might also be attributed to foci spatial dynamics, leading to foci merging in repair domains^59^, and this has been observed to depend also the genetic background^40^. Based on our dataset, it is not possible to discern if foci merging is due to damage proximity and superposition upon 2D projection or foci motion. Therefore, our modelling relies on geometrical parameters only, whose interpretation is hereafter recalled: *Δz* (microscope-dependent) is the thickness of the slice at focus; *r* is the *clustering radius*, related to foci physical extension, merging and resolution. What we learn from the modelling is that saturation of X-ray-induced foci yields with increasing dose can reproduced considering foci generated by initial DSBs, for different combinations of these geometrical parameters. In this case a good quantitative agreement with experimental data can be obtained, as it is observed in Fig. 6. To apply the same approach to foci data from the mixed neutron – photon field, we first performed transport calculations with PHITS to characterize the secondary charged species accelerated by neutrons, both in their relative dose contribution and dose mean lineal energy in biological samples (Table 1). For the species delivering most of the neutron dose (protons, ^12^C and ^16^O ions) we calculated with PARTRAC the associated DNA damage, when they have in the cell nucleus an average LET that is equal to ${\bar{{\rm{y}}}}_{{\rm{D}},{\rm{s}}}$ values given in Table 1. Predictions on dose-dependent readout of foci yields above background due to the different species found in the mixed neutron – photon field, but taken separately, are shown in Fig. 6. Such predictions were combined using dose weights to obtain the readout of foci yield after 1 Gy of the mixed neutron – photon field. By applying this coupling scheme, the expected foci yield is found to vary in the range 3 to 10 foci per cell, depending on the clustering parameters. Importantly, model predictions are close to experimental data varying the geometrical parameters used for the clustering algorithm in a range of values that are coherent with their interpretation: *Δz* is varied between 0.5–2 μm; *r* is varied between 0.5–1 μm. It has to be noted that the largest *Δz* = 2 μm value is not fully justified in light of the physical interpretation of this parameter as the DOF of the microscope. For X-ray-induced foci yields this parameter choice leads indeed to a significant overestimation of experimental data. This seems not to be the case for foci induced by the mixed neutron – photon field: a possible explanation is that foci from this field are brighter, and when focusing on the plane at the center of the nucleus, foci belonging at different depths can still appear in the image as halos bright enough to be scored (which is expected to have an impact only for earlier time-points/higher doses, with higher foci yields). It is also worth discussing how the parameter *r* relates to the distance parameter used in the combinatorial model developed in^59^. In their work, the authors hypothesize DSB motion and merging over large distances (μm). In the modelling, they assume that the nucleus is divided into separate repair domains, and DSBs induced in the same domain are merged into one single focus. The repair domain size (distance parameter) that allows the best reproduction of radiation-induced foci data (for both low- and high-LET radiation) is found to be of 1.5 μm. Following this approach, the *clustering radius r* that we use as a free model parameter could also be thought as implicitly simulating DSB motion assuming the existence of repair domains. However, based on a purely geometrical interpretation that takes into account at the same time foci physical extension and characteristics of the microscope, we want here to maintain the association between *r* and foci size: as already noted, *r* values are varied considering the size of foci observable in the image for the sham condition. Endogenous γ-H2AX foci in non-irradiated samples are small, homogeneously sparse in the nuclear volume and rare, hence at small risk of superposition upon 2D projection. Assuming a quasi-circular shape, their minimum radius has been found to be of 0.5 μm. The range of the *r* parameter has then been tentatively extended to 1 μm. As the dose increases, an increase in the average size of a focus when *r* is kept constant in the clustering algorithm would then reflect a higher number of foci in the selected slice, that merge upon 2D projection. With this interpretation, a value as high as 1.5 μm for the clustering radius would imply the detection of very large fluorescent areas, which seems not to be the case in our dataset even for the highest dose, for which instead, simple foci area definitions based on *r* seem to give a reasonable agreement with data^51^. For further developments of our or similar modelling approaches, a time-dependence of *r* (or of the equivalent distance parameters) could also be explored to mimic foci motion and merging, and its association with foci area might be dropped to allow for different interpretations of this parameter.

If we now limit only to the analysis of experimental data on foci yields at 30 minutes from the exposure, scored from 2D images, it is difficult to draw a quantitative conclusion on the biological effectiveness of the mixed neutron – photon field with respect to the low-LET reference field: the saturation of the signal with increasing dose prevents the adoption of the formal definition of RBE as ratio of doses inducing the same effect, and foci yields from the two fields are close if we consider an isodose comparison at 1 Gy: we measure on average ~7 and ~9 foci above background level per cell, respectively with the reference and the mixed neutron – photon field.

The modelling we propose here comes to help: at the same 1 Gy dose of the mixed field, we obtain (for the more realistic parameter combinations) a yield of foci in the range from ~6 per cell (*Δz* = 1; *r* = 0.5 μm) to ~10 per cell (*Δz* = 2; *r* = 0.5 μm), that includes the experimental value, and we know this corresponds to a higher damage yield (93.3 with respect to 46.9 DSBs Gy^−1^ per cell). As a first attempt, we might want to re-evaluate the effectiveness scoring foci in the whole nuclear volume instead than in a single cell slice. From an experimental point of view this can be done using either the *Z*-stack technique or resorting to confocal microscopy, both implying more time-consuming analysis for the higher number of images to be acquired and processed. However energy depositions from the mixed neutron – photon field under study in this work are as sparse in the cell volume as it is the case for a photon field, and there is no *a priori* reason to assume that detecting foci in the whole nucleus would lead to an improved evaluation of radiobiological effectiveness. Simulation results on foci reconstructed in 3D shown in Fig. 7 confirm this expectation: with the proposed coupling approach we obtain (*e*.*g*. for *R* = 0.5 μm) a predicted average yield of ~30 foci per cell at 1 Gy of the mixed field, to be compared to ~40 foci per cell for the same dose of the reference photon field. Moreover, predictions shown in Fig. 7 indicate that there is the possibility of signal saturation with increasing dose also for foci yields reconstructed in 3D, which would again make difficult the use of the formal definition of RBE. From these considerations, we conclude that it is difficult to draw a clear-cut conclusion on the radiobiological effectiveness of the mixed neutron – photon field (compared to a low-LET reference field) using as endpoint γ-H2AX foci induction, whether when scored from 2D images with conventional microscopy or in 3D in the whole nucleus. Information that can be obtained thanks to the modelling approach here proposed, as the average number of DSBs in a single focus, is essential to try to make quantitative statements.

From Tables 2 and 3 we get a confirmation that, if a similar foci yield would be scored in 3D for the mixed neutron – photon field with respect to the reference field, this would still correspond to a higher damage. For 1 Gy we have indeed that ~28 foci (*R* = 0.5 μm) account for the total yield of 93.3 DSBs in the mixed field, from which we derive an effective multiplicity of 3.3 DSBs per focus (in the range of DSB multiplicity values for secondary protons, that dominate the effectiveness of the field). The same dose of the reference field induce ~38 foci, accounting for 46.9 DSBs, with an effective multiplicity of 1.2 DSBs per focus. Eventually, multiplicity values obtained with our approach could be seen as “correction factors” to be applied to experimental data from 3D microscopy, to derive the information on the total damage and compare radiation effectiveness of different fields. The same could be said for the ratios of foci reconstructed in 2D and 3D, that could be seen as “scaling factors” to extrapolate from 2D microscopy data. It has to be noted that the 3D approach relies on the specific choice of the clustering radius *R* (as well as the 2D one relies on both *Δz* and *r*). In particular, DSB multiplicity values will depend on *R*, which, in turn, will change based on several biological factors, as *e*.*g*. chromatin condensation, cell type and the genetic of the strain.

In Fig. 8 we turn to the interpretation of data on residual foci yields at 24 hours from the exposure. The experimental foci yield from 2D images is compatible with the background level (zero foci above background) at 1 Gy for the X-ray field, and the same holds for the predicted foci yield from DSB clusters. At higher doses however, residual foci yields are significantly above background and linearly increase with increasing dose. Yields of foci from DSB clusters also slightly increase as a function of dose, but are always very low and become higher than the background level only for the highest 5 Gy dose and for the thickest nuclear slice (*Δz* = 1 μm). When a 3D reconstruction is applied, a net dose dependence for predicted foci yields is observed, but experimental results in 2D are even higher than model predictions for the 3D case. This might be ascribed to the oversimplified assumption of residual foci being the ones originating from DSB clusters. In particular, for a low-LET field, we do not expect a significant amount of complex damage: this is confirmed by model predictions of less than 1 DSB cluster Gy^−1^ for the reference X-ray field used in this work. Residual foci scored after exposure to doses higher than 1 Gy in this field indicate the presence of un-repaired damage after 24 hours, which is not exhausted by complex damage induction only. The “threshold” effect observed at 1 Gy might suggest that a failure of the DNA repair system happens at higher doses.

From Fig. 8 we also conclude that residual foci induction cannot be used as endpoint to quantify the effectiveness of the mixed neutron – photon field without the support of modelling: for the mixed and the reference fields, experimental yields at 1 Gy are compatible with zero above background. From the modelling we know instead that this translates in 3 to 4 vs. 0.5 foci from DSB clusters for the two fields in the whole nucleus. Interestingly, the modelling also suggests that the dose dependence of residual foci yields scored in 2D for the mixed field might be restored when going to 3D microscopy techniques, as it is the case for predictions on foci yields from DSB clusters obtained applying the 2D and 3D clustering algorithms.

To conclude, the approach presented in this work can assist the interpretation of measurements of radiation-induced DNA foci. Modelling is here used to go “behind” experimental observations: predictions are shown to be necessary to avoid mistaken conclusions on the relative effectiveness of a high-LET mixed neutron – photon field, when this is characterized using the γ-H2AX foci endpoint (foci yields above background level scored at 30 minutes after exposure), from 2D images obtained with ICC techniques and conventional fluorescence microscopy. An extension of the modelling to the 3D reconstruction of foci in the whole cell nucleus is also presented, which is necessary to integrate results from the burdensome image acquisition and analysis of foci with 3D microscopy techniques. Finally, the same approach is applied to interpret data of residual γ-H2AX foci yields (scored at 24 hours after exposure), using foci originating from DSB clusters (rather than from simple DSBs) as surrogate for unrepaired damage sites at late time-points. Though this assumption is certainly oversimplified, the modelling provides useful information, again necessary to avoid a wrong evaluation of radiobiological effectiveness.

This modelling approach can be extended to the study of any mixed field. In perspective, we could *e*.*g*. envisage the creation of a database with predictions of DNA repair foci yields induced as a function of particle type, energy, LET, irradiation condition, etc., to be queried for a quick assessment of the radiobiological effectiveness of a given test field, or, with further developments, for practical applications such as bio-dosimetry. Concluding, the adoption of this kind of approach, starting from radiation tracks and simulating the “observer”, can integrate experimental data, assist their interpretation, and also possibly drive the choice of techniques and measurements to be further performed, to achieve an unambiguous evaluation of the effectiveness of different radiation qualities.

## Data Availability

The datasets generated and/or analysed during the current study are available upon request from the corresponding author.
